# Enfermedad de Erdheim-Chester: primer caso pediátrico reportado en Colombia

**DOI:** 10.7705/biomedica.5651

**Published:** 2021-12-15

**Authors:** Luis Carlos Salazar, Luz Ángela Moreno, Lina Eugenia Jaramillo, Edgar Vladimir Cabrera

**Affiliations:** 1 Departamento de Radiología e Imágenes Diagnósticas, Facultad de Medicina, Universidad Nacional de Colombia, Bogotá, D.C., Colombia Universidad Nacional de Colombia Departamento de Radiología e Imágenes Diagnósticas Facultad de Medicina Universidad Nacional de Colombia Bogotá D.C. Colombia; 2 Unidad Funcional de Imágenes Diagnósticas, Fundación Hospital de La Misericordia, Bogotá, D.C., Colombia Unidad Funcional de Imágenes Diagnósticas Fundación Hospital de La Misericordia Bogotá D.C. Colombia; 3 Departamento de Patología, Facultad de Medicina, Universidad Nacional de Colombia, Bogotá, D.C., Colombia Universidad Nacional de Colombia Departamento de Patología Facultad de Medicina Universidad Nacional de Colombia Bogotá D.C. Colombia; 4 Laboratorio de Patología, Fundación Hospital de La Misericordia, Bogotá, D.C., Colombia Laboratorio de Patología Fundación Hospital de La Misericordia Bogotá D.C. Colombia; 5 Servicio de Oncohematología Pediátrica, Fundación Hospital de La Misericordia, Bogotá, D.C., Colombia Servicio de Oncohematología Pediátrica Fundación Hospital de La Misericordia Bogotá D.C. Colombia

**Keywords:** enfermedad de Erdheim-Chester, histiocitosis, pediatría, proteínas protooncogénicas B-raf, radiología, imagen por resonancia magnética, Erdheim-Chester’s disease, hypophysitis, pediatrics, B-Raf proto-oncogene proteins, magnetic resonance imaging

## Abstract

La enfermedad de Erdheim-Chester es una condición extremadamente rara en la edad pediátrica. Se presenta el caso de una niña de 12 años con diagnóstico histológico y radiológico de enfermedad de Erdheim-Chester multisistémica y mutación en el gen *BRAF*, que requirió tratamiento con dabrafenib.

Hasta el momento, se han reportado 22 casos pediátricos en el mundo y este es el segundo en Latinoamérica. Se observó el hallazgo radiológico denominado signo oscuro paraselar, descrito hasta ahora en pacientes con hipofisitis autoinmunitaria para diferenciarlos de aquellos con adenomas hipofisarios.

Este reporte contribuye a la literatura médica en dos aspectos fundamentales: las manifestaciones clínicas de la enfermedad y su diagnóstico en la población pediátrica.

## Abstract (en)

The Erdheim-Chester’s disease is extremely rare in children. We present the case of a 12-year-old girl with histological and radiological diagnosis of this disease and mutation of the *BRAF* gene, who developed multisystemic compromise requiring treatment with dabrafenib. We identified 22 reports of this condition among children worldwide and this is the second pediatric case in Latin America. Diagnostic imaging is critical to confirm Erdheim-Chester disease and for the surgical planning of the biopsy. Additionally, we identified the parasellar dark sign, which has previously been reported on lymphocytic hypophysitis.

This report contributes to the current practice as it shows the clinical presentation and the diagnostic workout of this disease in pediatrics.

La enfermedad de Erdheim-Chester hace parte del grupo de las histiocitosis, las cuales se caracterizan por ser trastornos sistémicos en los cuales hay una proliferación descontrolada de células derivadas del sistema mononuclear fagocítico (células dendríticas, monocitos y macrófagos), que infiltran diferentes órganos del cuerpo ^1^. Recibe su nombre gracias a la publicación realizada en 1930 por los patólogos Jakob Erdheim (Austria) y William Chester (Estados Unidos) ^2^. Clásicamente, se la describe como una histiocitosis de fagocitos mononucleares distintos a las células de Langerhans y pertenece al grupo II según la clasificación del *Working Group of the Histiocyte Society* de 1987 ^3^. En la clasificación de la *Histiocyte Society* de 2016 corresponde al grupo L, o grupo Langerhans, que reúne la histiocitosis de células de Langerhans, la histiocitosis de células indeterminadas, la enfermedad de Erdheim-Chester y la mixta, de histiocitosis de células de Langerhans más enfermedad de Erdheim-Chester ^1^.

A pesar de ser una histiocitosis, se considera una condición extremadamente infrecuente en la edad pediátrica, siendo más común en la adultez ^4^; es parte del grupo de las enfermedades raras, según la *European Rare Disease Organization* y la *National Organization for Rare Disorders*. En el 2014, el número total de casos reportados fue de 550, la gran mayoría de ellos diagnosticados en la última década, cifra que sigue aumentando debido al reconocimiento de la enfermedad ^5^.

Oskaya, *et al.,* reportaron mutaciones en los genes *BRAF* en el 65 % de los pacientes con esta condición y en el gen *MAP2K1* en el 16 %. Estos hallazgos, junto con las mutaciones en otras proteínas cinasas activadas por mitógenos, sugerirían la naturaleza tumoral de la enfermedad y su clasificación como una neoplasia histiocitaria. Sin embargo, en la actualidad la enfermedad de Erdheim-Chester se considera una condición benigna de naturaleza inflamatoria ^6^.

El síntoma más frecuentemente descrito en esta enfermedad es el dolor óseo; asimismo, la diabetes insípida hace parte de su presentación habitual, tal como ocurre en la histiocitosis de células de Langerhans. La enfermedad de Erdheim-Chester puede cursar casi sin síntomas cuando el compromiso es predominantemente cutáneo u óseo, o ser sintomática, si predomina el compromiso del sistema nervioso central, cardiaco, retroperitoneal, órbito- craneo-facial, neuroendocrino, pulmonar o multisistémico ^4^.

El diagnóstico se hace con base en parámetros histológicos y radiológicos. Histológicamente, se caracteriza por la presencia de histiocitos cargados de lípidos con aspecto xantomatoso en un fondo de fibrosis. Cuando se ven células gigantes de Touton, la enfermedad es indistinguible del xantogranuloma juvenil, motivo por el cual los hallazgos clínicos y radiológicos son fundamentales para establecer el diagnóstico definitivo ^4^. Estas células son reactivas en la inmunohistoquímica para CD68, CD163 y el factor XIIIa, y negativas para CD1a y langerina (CD207), en tanto que el marcador S100 tiene reactividad variable. En la ultraestructura, carecen de gránulos de Birbeck.

La enfermedad se caracteriza por un compromiso osteoesclerótico metafisiario y simétrico de los huesos largos, tanto en la tomografía como en la resonancia magnética, lo cual a menudo no se observa en las proyecciones radiográficas debido a su menor sensibilidad. Existe una fuerte captación metafisiaria en la gammagrafía ósea con Tc-99m. Sin embargo, el 4 % de los pacientes con enfermedad de Erdheim-Chester no presenta el compromiso óseo radiológico clásico, por lo que en esos casos es necesario basarse en la histopatología y en otros órganos usualmente involucrados (4).

La enfermedad es de mal pronóstico, con una supervivencia de 32 meses en el 57 % de los casos ^7^, y es de peor pronóstico cuando existe compromiso cardiaco o del sistema nervioso central. No obstante, la supervivencia ha aumentado con el advenimiento de los nuevos tratamientos. El medicamento de primera línea es el interferón alfa y la segunda línea incluye el vemurafenib, infliximab, anakinra o el dabrafenib ^8^^,^^9^.

## Presentación del caso

Se trata de una niña de 12 años que consultó por dolor abdominal, ictericia, coluria, acolia y prurito generalizado asociado con pérdida de peso (3 kg en un mes). Entre los antecedentes, se registró el nacimiento a término sin complicaciones, con bajo peso al nacer para la edad gestacional (2.400 g), adecuado desarrollo psicomotor y buen rendimiento académico. En la consulta inicial, se evidenció baja talla para la edad (desviación estándar de -3,58) y ausencia de signos de desarrollo sexual secundario como pubarquia o telarquia. Además, la paciente refirió polidipsia, poliuria y dolor osteoarticular desde los primeros años de vida.

En el examen físico de ingreso, no se registró alteración de los signos vitales y la presión arterial era normal para la edad; se detectó ictericia, no había adenomegalias en el cuello, y el abdomen era blando y depresible, con leve dolor en el hipocondrio derecho. En el examen neurológico, no se encontró alteración de la esfera mental ni déficit cognitivo, el lenguaje era fluido, los reflejos oculomotores bilaterales estaban conservados y los movimientos oculares eran simétricos y sin restricción, aunque había disminución de la agudeza visual en el ojo izquierdo y nistagmo multidireccional con la mirada extrema derecha; no se registraba déficit motor o alteración de los reflejos musculotendinosos en las cuatro extremidades, había reflejo plantar neutro bilateral, sin signos cerebelosos ni alteraciones de la marcha.

En los exámenes de laboratorio no se encontraron alteraciones hematológicas, como coagulopatías, ni elevación de la concentración sérica del complemento; entre los reactantes de fase aguda, la velocidad de sedimentación globular (VSG) estaba elevada, en tanto que el valor de la proteína C reactiva era normal. La paciente presentaba elevación del sodio sérico con densidad urinaria disminuida, pero sin alteración de la función renal. Se observó aumento de la gamma-glutamil transferasa (GGT), de la fosfatasa alcalina y de la bilirrubina conjugada; las concentraciones de alanino aminotransferasa (ALT) y de aspartato aminotransferasa (AST), eran normales. Se comprobó que había hipercolesterolemia y no hubo alteración de las proteínas séricas. No se observaron cambios de infección reciente en los exámenes específicos practicados. Se demostró panhipopituitarismo hipogonadotrófico con hipotiroidismo central e hipocortisolismo (cuadro 1).

**Cuadro 1:** Resultados de las pruebas de laboratorio

Hematológicas		Función hepática		Perfil infeccioso	
Prueba	Resultado	Prueba	Resultado	Prueba	Resultado
Leucocitos	10.730 por ml	ALT	83,6 U/L	IgG toxoplasma	Negativo
Hemoglobina	11,5 g/dl	AST	98,8 U/L	IgM toxoplasma	Negativo
Plaquetas	261.000 por mm^3^	GGT**	724,2 U/L	IgG citomegalovirus	Positivo
INR	0,94	LDH	85 U/L	IgM citomegalovirus	Negativo
C3	171 mg/dl	FA**	1.682 mg/dl	IgG Epstein-Barr	Positivo
C4	27 mg/dl	Albúmina	3,1 g/dl	IgM Epstein-Barr	Negativo
Glucosa	84 mg/dl	Bilirrubina total**	4,3 mg/dl	IgM HAV	Negativo
Lactato	1,2 mmol/L	Bilirrubina directa**	4,2 mg/dl	AgHBs	Negativo
VSG**	56 mm/hora	Función renal		Anti-HCV	Negativo
PCR	16 mg/L	Prueba	Resultado	RPR	Negativo
Electrolitos		Creatinina	0,34 mg/dl	Endocrinológicos	
Prueba	Resultado	BUN	5,2 mg/dl	Prueba	Resultado
Sodio**	147 mEq/L	Perfil lipídico		TSH	1,76 mU/L
Potasio	3,7 mEq/L	Prueba	Resultado	T4L*	0,595 ng/dl
Cloro	109 mEq/L			IGF1	< 15 ng/ml
Calcio	8,7 mEq/L	Colesterol total**	871 mg/dl	Prolactina	46 ng/ml
Uroanálisis		HDL**	16 mg/dl	FSH*	0,1 mUI/ml
		LDL**	784 mg/dl	LH*	0,1 mUI/ml
Prueba	Resultado	Triglicéridos**	169 mg/dl	Estradiol	< 10 pg/ml
Densidad*	1.005			Cortisol*	4,9 mcg/dl

INR: relación normalizada internacional; C3: componente 3 del complemento; C4: componente 4 del complemento; VSG: velocidad de sedimentación globular; PCR: proteína C reactiva; ALT: alanina aminotransferasa; AST: aspartato aminotransferasa; GGT: gamma-glutamil transferasa; LDH: lactato deshidrogenasa; FA: fosfatasa alcalina; BUN: nitrógeno ureico; HDL: lipoproteína de alta densidad; LDL: lipoproteína de baja densidad; Ig: inmunoglobulina; HAV: virus de la hepatitis A; AgHBs: antígeno de superficie del virus de la hepatitis B; Anti-HCV: anticuerpos contra hepatitis C; RPR: reagina plasmática rápida; TSH: hormona estimulante de la tiroides; T4L: tiroxina libre; IGF1: factor de crecimiento insulínico de tipo 1; FSH: hormona foliculoestimulante; LH: hormona luteinizante.

* Valor disminuido

** Valor elevado

Los estudios electrofisiológicos mostraron potenciales evocados auditivos anormales indicativos de hipoacusia neurosensorial leve izquierda y potenciales visuales normales con integridad de la vía retinocortical.

**Figura 1:**
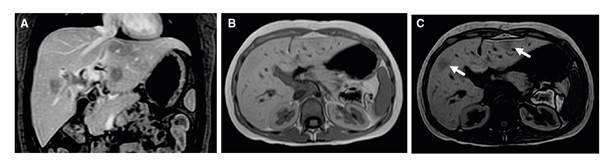
Resonancia magnética abdominal que evidencia lesiones hepáticas hipovasculares con contenido graso en T1 contrastado, con supresión grasa **(A**), en fase (**B**) y fuera de fase (**C**)

La resonancia magnética abdominal evidenció el compromiso multifocal del hígado, con lesiones sólidas de localización aleatoria, hipointensas en T1 e isointensas en T2, hipovasculares, con caída de la señal en secuencias fuera de fase y medidas entre 9 y 20 mm. Había dilatación de las vías biliares intrahepática y extrahepática, y el colédoco tenía un diámetro de 7,5 mm (figura 1).

**Figura 2:**
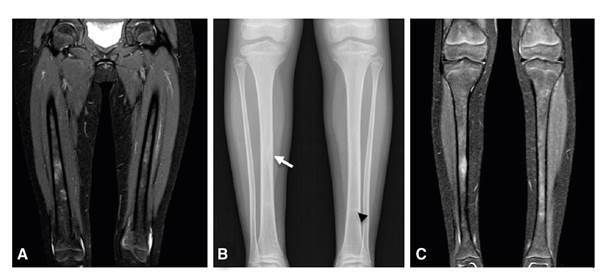
Extremidades inferiores. **A.** Resonancia magnética: engrosamiento cortical en el fémur. **B.** Radiografía simple: aspecto festoneado endóstico sin reacción perióstica (flecha) en las tibias y lesión esclerótica (punta de flecha) izquierda **C.** Resonancia magnética: patrón parcheado en la médula ósea, con lesiones focales hipointensas en T1 e hiperintensas en STIR, en las diáfisis tibiales y femorales, y en la zona metadiafisiaria proximal de la tibia izquierda.

En la radiografía de miembros inferiores, se observó un festoneado endóstico focal en la diáfisis de la tibia derecha, pero sin reacción perióstica. En la resonancia magnética se apreció un patrón parcheado en la médula ósea de las extremidades inferiores, con lesiones focales hipointensas en T1 e hiperintensas en STIR, en las diáfisis tibiales y femorales, y en la zona metadiafisiaria proximal de la tibia izquierda; no se demostró compromiso de los tejidos blandos (figura 2).

**Figura 3:**
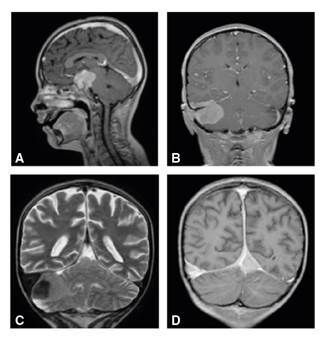
Resonancia magnética cerebral: **A**. en plano sagital, lesión selar extraxial con marcado realce en la secuencia potenciada en T1 contrastado y aumento del realce de los senos durales; **B**. lesión infratentorial en plano coronal; **C**. la misma lesión con baja señal en T2 y aumento del tamaño del seno transverso derecho; **D**. infiltración de la tórcula y el seno transverso derecho, así como engrosamiento dural bilateral en T1 contrastado.

En la resonancia magnética cerebral se observaron dos masas extraxiales de bordes bien definidos, hipointensas en T1 y T2, con marcado realce. La primera masa era de localización selar y paraselar, con compromiso de los senos cavernosos, estenosis grave de las arterias carótidas internas y cerebrales posteriores, y compresión del quisma óptico y de la glándula pituitaria. La segunda masa era infratentorial derecha, con compresión del hemisferio cerebelar del mismo lado, pero sin efecto compresivo sobre el tallo cerebral ni el cuarto ventrículo. No se observó compromiso de los pares craneanos. Se evidenció engrosamiento de la duramadre en el tentorio y la hoz del cerebro, y además, dilatación e infiltración de los senos durales, el transverso derecho y la tórcula (figura 3).

**Figura 4:**
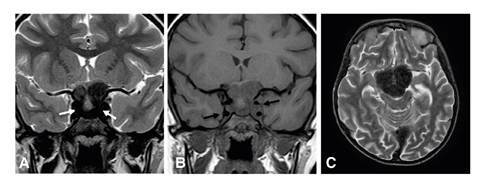
Resonancia magnética cerebral. **A**. el signo oscuro (flechas blancas) en secuencias potenciadas en T2 coronal y **C**. axial; **B**. segmento cavernoso de las arterias carótidas internas en T1 en el plano coronal (flechas negras)

Asimismo, se observó hipointensidad en las secuencias T2 en las regiones selar y paraselar, hallazgo correspondiente al signo oscuro o *dark sign*, es decir, baja intensidad de la señal, similar a la de la cortical ósea alrededor de la hipófisis y dentro de los senos cavernosos; este signo ha sido descrito en la hipofisitis autoinmunitaria y permite diferenciarla del adenoma hipofisario ^10^ (figura 4).

**Figura 5:**
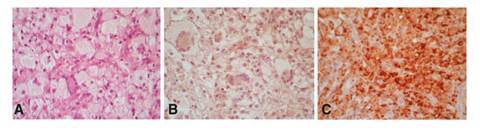
A. Duramadre infiltrada por histiocitos xantomatosos de tamaño variable, con células gigantes, algunas con múltiples núcleos que forman un anillo (células de Touton). Hematoxilina y eosina, 40X. B. Inmunohistoquímica con CD1a que muestra negatividad de las células xantomatosas con la tinción de membrana (expresión débil inespecífica para citoplasma), 40X. C. Inmunohistoquímica con CD68 que evidencia reactividad de las células xantomatosas, 40X.

En la biopsia intracraneal, se observó la infiltración por una lesión xantomatosa con histiocitos de tamaños variados mezclados con células de Touton, en músculo, hueso y duramadre (figura 5A). La inmunohistoquímica fue negativa para el patrón de membrana para CD1a, marcando en forma débil y heterogénea el citoplasma, y fue reactiva para CD68 (figura 5B-C). Algunas células mostraron reactividad variable (fuerte o débil) para el S100, por lo que se planteó que podría tratarse de una histiocitosis no Langerhans o una dislipidemia con xantomas asociados (figura 5).

Se practicó una biopsia hepática en la que se observó el hígado nodular con puentes fibrosos edematosos, proliferación de los conductos y abundantes macrófagos que predominaban en la interfase lesionando la placa limitante, con reacción positiva para CD68 y negativa para CD1a; no se observaron células xantomatosas ni de tipo Touton. Con la correlación histológica, se sugirió que podría tratarse de una enfermedad de Erdheim-Chester (cuadro 2).

**Cuadro 2:** Resultados de las pruebas genéticas y de inmunohistoquímica

Inmunohistoquímica		Genética	
Prueba	Resultado	Prueba	Resultado
CD68	Acentuadamente positivo	Mutación BRAF V600E	Positiva heterocigota
S100	Ocasionales células reactivas		
CD1a	Negativo		
CD45	Positivo		
Ki67	Varios leucocitos en proliferación		

En las pruebas genéticas, se encontró la mutación *BRAF* V600E, por lo que se inició la administración de 50 mg de dabrafenib cada 12 horas por vía oral; inicialmente, la paciente toleró adecuadamente el tratamiento (cuadro 2).

### Consideraciones éticas

Se obtuvo autorización escrita del acudiente de la paciente para usar la información clínica y las pruebas diagnósticas descritas en esta publicación.

## Discusión

Para establecer la situación actual y los casos reportados de la enfermedad, se hizo una búsqueda sistemática en Medline (Ovid) y LILACS, tanto con lenguaje controlado como con texto libre (anexo 1). Se encontraron 66 referencias en las cuales se evaluaron el título, el resumen y el texto completo para seleccionar los reportes de caso o series de casos de la enfermedad de Erdheim-Chester en pacientes menores de 18 años: 19 artículos cumplían con los criterios de inclusión ^7^^,^^9^^,^^11^^-^^31^. Se excluyeron 47, de los cuales cinco correspondían a series de casos de enfermedad de Erdheim-Chester que no discriminaban entre adultos y niños. El total de casos reportados fue de 22 (cuadro 3). Los estudios se habían desarrollado en Asia (11 casos), Europa (7 casos) y América (4 casos). La edad de los pacientes fluctuaba en un rango entre 1,7 y 17 años, con un promedio de 9 años; 11 casos fueron en niños y 11 en niñas. En todos los pacientes se estableció el compromiso óseo, seguido por el del sistema nervioso central en 55 % de ellos. Se reportó también compromiso orbitario, cutáneo, abdominal, pulmonar, renal, pleural, mediastinal y retroperitoneal.

**Cuadro 3:** Caracteristicas de los casos

Año	País	Edad (años)	Sexo	Extensión de la enfermedad	Histología	BRAF	Tratamiento	Resp.	Seg.	Com.	Ref.
2019	Colombia	12	F	SNC, OSE, ABD	EEC	+	Dabrafenib	Sí	1A	Ninguna	RA
2018	China	11	M	SNC, OSE, ORB	HCL/EEC	+	Cirugía, dabrafenib	Sí	NR	NR	(9)
2018	China	3,5	M	OSE, CUT	EEC	-	INTa/2a	Sí	NR	NR	(12)
2017	Hungría	1,7	M	OSE, CUT	HCL/EEC	+	Vemurafenib (3a línea)	Sí	2A	NR	(13)
2016	India	6	F	OSE	EEC	-	NR	NR	NR	LLA	(14)
2016	EUA	15	M	SNC, OSE	EEC	NR	Anakinra, vinblastina	Sí	NR	NR	(15)
2016	CdS	3	M	SNC, OSE, ORB	HCL/EEC	+	Cladribina, citarabina (2a línea)	Sí	NR	NR	(16)
2016	EUA	7	M	SNC, OSE	EEC	+	Anakinra (4a línea)	Sí	2A	NR	(17)
2015	Irán	14	M	SNC, ORB	EEC	NR	Cirugía, citotóxico, CS	Sí	8A	NR	(18)
2014	Singapur	14	F	SNC, OSE	EEC	NR	INTa	Sí	1A	LLA, DM	(19)
2014	México	2	F	OSE, ORB	EEC	NR	Vinblastina, CS (2a línea)	Sí	5A	NR	(11)
2012	China	11	F	OSE	EEC	NR	INTa	Sí	4M	NR	(20)
2012	CdS	4	M	SNC, OSE, PUL	EEC	NR	INTa/2a, ciclosporina, CS (2a línea)	Sí	6M	NR	(21)
2011	China	11	F	OSE	EEC	NR	La familia rechaza el tratamiento	NR	NR	NR	(22)
2010	Francia	17	M	OSE, ORB, REN	EEC	NR	NR	NR	NR	NR	(23)
2010	Francia	6	F	SNC, OSE, REN, PLE	EEC	NR	INTa	Sí	NR	NR	(23)
2009	Francia	10	F	OSE, RET	EEC	-	Canakinumab (3a línea)	Sí	5A	NR	(24-26)
2007	Turquía	10	M	SNC, OSE	EEC	NR	CS	NR	NR	NR	(27)
2005	Japón	13	F	SNC, OSE, ABD	EEC	NR	NR	NR	NR	NR	(28)
2004	CdS	10	F	OSE	EEC	NR	CS	Sí	3A	NR	(29,30)
2003	Italia	14	F	SNC, OSE, ABD	EEC	NR	Carboplatino, etopósido (2a línea)	+/-	3A	NR	(31)
1991	EUA	7	M	SNC, OSE, MED, RET	EEC	NR	NR	NR	2,5A*	NR	(7)
1991	Francia	17	F	OSE, ORB	EEC	NR	CS	NR	2,5A*	NR	(7)

EUA: Estados Unidos de América; CdS: Corea del Sur; SNC: sistema nervioso central; OSE: óseo; ORB: orbitario; CUT: cutáneo; PUL: pulmonar; REN: renal; PLE: pleural; RET: retroperitoneal; ABD: abdominal; MED: mediastinal; BRAF: mutación BRAF V600E; NR: no reporta; RA: reporte actual; INTα: interferón alfa; CS: corticoesteroide; A: años; M: meses; LLA: leucemia linfoide aguda; DM: diabetes mellitus; Resp.: respuesta al tratamiento; Seg.: seguimiento; Com.: comorbilidades; Ref.: referencias; EEC: enfermedad de Erdheim-Chester; HCL/EEC: histiocitosis de células de Langerhans más enfermedad de Erdheim-Chester.

El diagnóstico se confirmó mediante histología e inmunohistoquímica en todos los casos. Por histopatología, se confirmaron 19 casos de enfermedad de Erdheim-Chester sola y tres de la mixta, de histiocitosis de células de Langerhans más enfermedad de Erdheim-Chester. Además, en siete de los casos se estudió la posible mutación V600E del gen *BRAF*, la cual se confirmó en cuatro (4/7, 57 %) de ellos.

El tratamiento se reportó en 17 de los casos y, en siete de estos pacientes, fue necesario utilizar más de un tipo de tratamiento. Los esquemas terapéuticos definitivos incluyeron: inmunosupresores como corticoesteroides en seis casos y ciclosporina en uno, interferón alfa en cinco, antagonistas del receptor de interleucina en uno, anakinra en dos y canakinumab en uno; además, se usó quimioterapia citotóxica en cinco casos, inhibidores de la enzima asociada al *BRAF* en dos y cirugía en otros dos. La respuesta al tratamiento se consideró adecuada en 14 de los 15 (93 %) pacientes en quienes se reportó, en tanto que en el otro caso la respuesta fue parcial. El seguimiento de los pacientes se describió en 12 casos, con una media de tiempo de 2,8 años. Se reportó la muerte de dos pacientes a los 2,5 años del diagnóstico de la enfermedad.

En el presente caso, se presentaron los dos tipos de compromiso más frecuentes en los pacientes pediátricos: el óseo, descrito en el 100 % de los casos, y el del sistema nervioso central, descrito en el 55 % de ellos. A diferencia de lo reportado en la literatura, en esta paciente la presentación clínica fue un síndrome ictérico debido al compromiso hepático confirmado histológicamente.

Las imágenes diagnósticas no solo fueron indispensables para detectar el compromiso óseo, esencial en el diagnóstico de la enfermedad de Erdheim-Chester, sino también, las lesiones hepáticas y cerebrales (infiltración de los senos durales), lo que contribuyó a la planeación y la toma de las biopsias. Además, se detectó el hallazgo radiológico denominado signo oscuro paraselar o *dark sign*, observado en las secuencias T2 (figura 2), el cual consiste en la baja intensidad de la señal alrededor de la hipófisis y dentro de los senos cavernosos que, hasta el momento, se ha descrito en pacientes con hipofisitis autoinmunitaria para diferenciarlos de aquellos con adenomas hipofisarios ^10^.

Este es el primer caso de enfermedad de Erdheim-Chester en la población pediátrica reportado en Colombia y el segundo en Latinoamérica. En Colombia, se había reportado un caso de esta enfermedad en un adulto de 53 años con diagnóstico de fractura patológica de húmero ^32^. En Latinoamérica, se había reportado un caso en México, el de una niña de dos años de edad con compromiso óseo y orbitario, cuya reacción terapéutica a la quimioterapia citotóxica de segunda línea con vinblastina y corticoesteroides fue adecuada ^11^.

## Conclusión

La enfermedad de Erdheim-Chester es una condición extremadamente rara en la infancia, cuya presentación es heterogénea y multisistémica. Para lograr un diagnóstico adecuado, se deben tener en cuenta los hallazgos clínicos, los histopatológicos y los radiológicos. Los reportes de casos de la enfermedad permiten conocerla mejor y, por ende, diagnosticarla y tratarla de forma adecuada.

## Archivos suplementarios

**Anexo 1.** Estrategia de búsqueda

Base de datos: Ovid MEDLINE(R) ALL <1946 to January 25, 2019>

Estrategia de búsqueda:

1. exp Erdheim-Chester Disease/ (543)
2. Erdheim-Chester.tw. (787)
3. 1 or 2 (858)
4. exp Child/ (1807369)
5. exp Child, Preschool/ (869587)
6. exp Infant/ (1085169)
7. Infant, Newborn {Incluyendo términos relacionados} (11423)
8. (Child* or Preschool* or Infant* or Newborn*).tw. (1628524)
9. pediatr*.tw. (260617)
10. or/4-9 (2855739)
11. 3 and 10 (49)
